# Distribution and nests of paper wasps of *Polistes* (*Polistella*) in northeastern Vietnam, with description of a new species (Hymenoptera, Vespidae, Polistinae)

**DOI:** 10.3897/zookeys.368.6426

**Published:** 2014-01-08

**Authors:** Lien Thi Phuong Nguyen, Jun-ichi Kojima

**Affiliations:** 1 Insect Ecology Department, Institute of Ecology and Biological Resources, Vietnam Academy of Science and Technology, 18 Hoang Quoc Viet Road, Nghia Do, Cau Giay, Hanoi, Vietnam; 2 Natural History Laboratory, Faculty of Science, Ibaraki University, Mito, 310−8512 Japan

**Keywords:** Hymenoptera, Vespidae, Polistinae, *Polistes*, *Polistella*, new species, nest, northeastern Vietnam

## Abstract

Seven species of the subgenus *Polistella* Ashmead of the genus *Polistes* Latreille including a new species, *P. brunetus* Nguyen & Kojima, **sp. n.** described here, are recognized to occur in northeastern Vietnam, the easternmost part of the eastern slope of the Himalayas. A key to these species is provided. Their distributional records are remarked. Nests of *P. delhiensis* Das & Gupta, *P. mandarinus* de Saussure and *P. brunetus* are also described.

## Introduction

Of the four subgenera in the cosmopolitan paper wasp genus *Polistes*, *Polistella*, with some 85 extant species, is the largest in terms of the number of species among the three subgenera endemic to Old World (*Gyrostoma* Kirby & Spence, *Polistella* Ashmead, and *Polistes* Latreille). The subgenus *Polistella* is known to show a high species diversity in the northern part of Indochina, the area on the eastern slope of the Himalayas. This is especially the case, together with strong endemism, for the *Polistella* species that are characterized by a basally strongly swollen second metasomal sternum. These species may form a monophyletic group and show the distribution pattern of so−called “Himalayan Corridor origin”, namely they occur in the zone from the southern slopes of the Himalayas, through the eastern slope of the Himalayas and eastern coastal areas of continental Asia and Taiwan, to Ussuri and eastern Siberia in Russia and Hokkaido in Japan (Nguyen et al. 2011). Locating in the easternmost part of the eastern slope of the Himalayas, the *Polistella* fauna in the northern parts of Vietnam would be a key to understanding the process of forming the current distribution pattern of these *Polistella* wasps.

While the *Polistella* fauna of northwestern Vietnam has been more or less well studied (Nguyen et al. 2011), that in northeastern Vietnam has been little known. The present study has recognized seven species of *Polistes (Polistella)* including a new species described herein to occur in northeastern Vietnam. Their distribution records are remarked. Nests of three species (*Polistes delhiensis* Das & Gupta, *Polistes mandarinus* de Saussure and *Polistes brunetus* Nguyen & Kojima, sp. n.) are also described.

## Materials and methods

**Figure 1.**
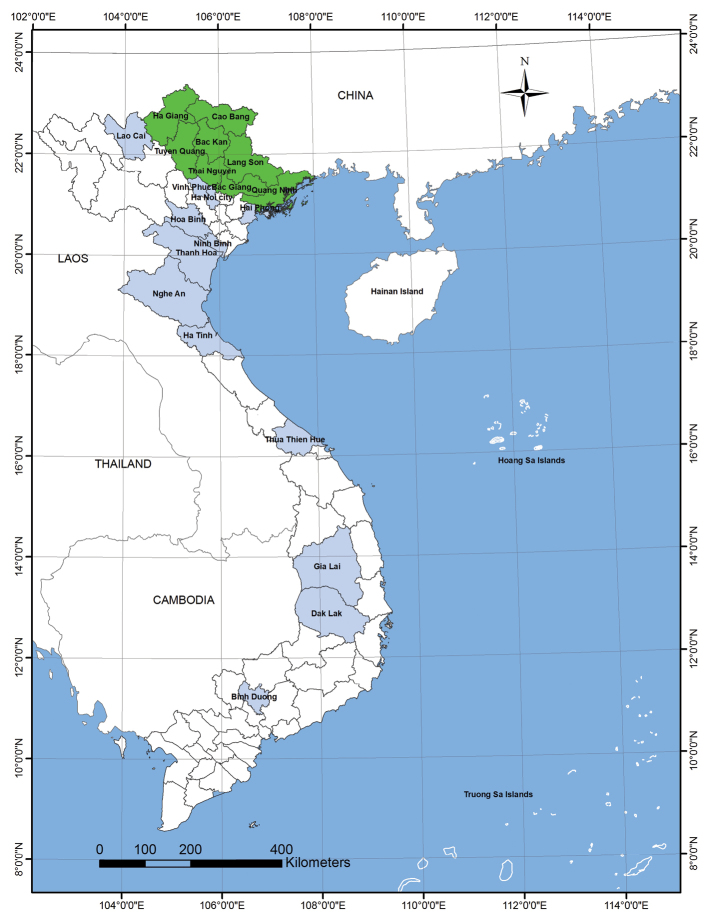
Map of Vietnam showing the provinces in the northeastern part (green) and those in which the specimens examined were collected (light violet).

Based on the geographical and climatic features, “northeastern Vietnam” is used in the present paper for the area consisting of the following provinces: Ha Giang, Cao Bang, Tuyen Quang, Bac Kan, Thai Nguyen, Lang Son, Bac Giang and Quang Ninh (Fig. 1). The specimens examined in the present study are unless otherwise mentioned deposited in the Institute of Ecology and Biological Resources in Hanoi; they were mainly collected by ourselves during a research trip to Cao Bang, Bac Kan and Bac Giang made in 2012.

The adult morphological and color characters except for male terminal sterna and genitalia were observed on pinned−and−dried specimens under a stereomicroscope. Apical parts of male metasomata were dissected for the terminal sterna and genitalia. They were put in lactic acid for several hours, washed in distilled water, and observed in glycerin under a stereomicroscope. The terminology of male genitalia follows Kojima (1999). Drawings were made with the aid of a drawing tube. Photos were taken with Panasonic Lumix DMC−FX 100 and Leica EZ4HD 3.0 MegaPixel Digital Stereo Microscope, using LAS exclusive microscopy software (LAS EZ 2.0.0).

In the descriptions of morphology, the following abbreviations are used: POD, distance between the inner margins of the posterior ocelli; OOD, distance between the outer margin of the posterior ocellus and the inner margin of the eye at vertex; Od, transverse diameter of the posterior ocellus.

The parts measured for the morphometric are defined as follows: body length, the lengths of head, mesosoma and first two metasomal segments combined; clypeus width, the distance between the uppermost points where clypeus touches the eyes; clypeus height, the distance from the bottom of the dorsal emergination to the apex; distance between inner eye margins at vertex and at clypeus, respectively the distance between the inner eye margins at the level of anterior ocellus in frontal view of head and at the level where inner eye margins approached each other most closely; interantennal and antennocular distances, the distance between the inner margins of antennal sockets and between the outer margin of antennal socket and inner eye margin at the level of middle of antennal socket, respectively; antennal socket width, the transverse diameter; eye and gena width, the maximum width for each in strictly lateral view of the head; metasomal tergum I length, the distance in lateral view from the posterior end of the basal slit for the reception of the propodeal suspensory ligament to the posterodorsal end of the tergum; metasomal tergum II, length, the distance in lateral view from the bottom of the basal depression or “neck” to the posterodorsal end of the tergum; metasomal tergum I and II width, the maximum width for each in dorsal view.

## Taxonomy and distribution

### Polistes (Polistella) dawnae

Dover & Rao, 1922

http://species-id.net/wiki/Polistes_dawnae

Polistes dawnae Dover & Rao, 1922: 248, female, Dawna Hills, Burma [Myanmar], [holotype in the Zoological Survey of India, Calcutta].

#### Material examined.

Northeastern provinces: **Bac Kan:** 2 females, Kim Hy NP, Na Ri, 22°14'N, 106°05'E, alt. ca 300 m, 3.VIII.2012, L.T.P. Nguyen et al. Other province: **Son La:** 1 female, Nong Truong, Moc Chau, 20°50'N, 104°40.283'E, alt. ca 950 m, 2.VII.2013, D.D. Nguyen.

#### Remarks on distribution.

*Polistes dawnae*, one of the two species characterized by a basally strongly swollen second metasomal sternum and recorded in northeastern Vietnam (the other is *Polistes mandarinus*), was originally described from Dawna Hills [16°50'N, 98°15'E], northern Myanmar, and has been recently recorded in northeastern part of Laos (Gusenleitner 2013) and northern Vietnam, such as in the provinces of Lai Chau, Dien Bien, Hoa Binh (Nguyen et al. 2011), Bac Kan and Son La (present study). This species may be restricted in its distribution to the areas on the eastern slope of the Himalaya.

### Polistes (Polistella) mandarinus

de Saussure, 1853

http://species-id.net/wiki/Polistes_mandarinus

Polistes mandarinus de Saussure 1853 in de Saussure 1853−1858: 58, female, “Le norde de la Chine” [lectotype in The Natural History Museum, London].

#### Material examined.

Northeastern provinces: **Cao Bang:** 12 males, 4 females, Phi Oac Nature Reserve, Thanh Cong, Nguyen Binh, 22°32.5'N, 105°53'E, alt. ca 1000 m, L.T.P. Nguyen et al. [11 males, 3 females, Nest#VN−NE2012−P−07, 8.VIII.2012; 1 male & 1 female, 9.VIII.2012]; 2 females, Phi Oac Nature Reserve, 22°35.567'N, 105°51.417'E, alt. ca 1035 m, 7−10.V.2013, T.V. Hoang. **Bac Kan:** 7 females, Kim Hy NP, Na Ri, alt. ca 600−700 m, 22°19'N, 105°54'E, Nest#VN−NE2012−P−03, 4.VIII.2012, L.T.P. Nguyen et al. Other province: **Hai Phong:** 1 female, Cat Ba NP, Cat Hai, 20°47'N, 106°59'E, 26.VII.2013, L.T.P. Nguyen & D.D. Nguyen.

#### Remarks on distribution records.

The distribution records of *Polistes mandarinus* reported may need confirmation as several species were erroneously identified as “*Polistes mandarinus*” (see Kojima 1997). In Vietnam, this species has been known from the provinces of Quang Tri (Nguyen and Ta 2008), Phu Tho, Vinh Phuc, Thua Thien Hue (Nguyen et al. 2011), Cao Bang, Bac Kan, Hai Phong (present study); this species may occur in the areas north of the Hai Van Pass, but its occurrence in nothwestern Vietnam needs further researches. The species has also been recorded from eastern China and Tibet (Hou et al. 2012) and Korea (Carpenter 1996), however its occurrence in Korea may need confirmation (J.K. Kim & J. Kojima, unpublished data).

### Polistes (Polistella) delhiensis

Das & Gupta, 1989

http://species-id.net/wiki/Polistes_delhiensis

Polistes delhiensis Das & Gupta, 1989: 63, female, Delhi [India], [holotype in Zoological Survey of India, Calcutta].

#### Material examined.

Northeastern provinces: **Ha Giang:** 1 female, Cao Bo, Vi Xuyen, 22°44'N, 104°54'E, alt. ca 532 m, 21−24.IV.2000, L.T.P. Nguyen; **Bac Kan:** Kim Hy NP, Na Ri, 22°14'N, 106°05'E, alt. ca 600−700 m, 4.VIII.2012, L.T.P. Nguyen et al. [1 female, 3 females of Nest# VN−NE2012−P−04]; 2 females, Kim Hy NP, Vu Muon, Bach Thong, alt. ca 550 m, 22°12.5'N, 105°58'E, 5.VIII.2012, L.T.P. Nguyen et al. Other provinces: **Phu Tho:** 2 females, Xuan Son NP, 21°10'N, 104°58'E, alt. ca 400 m, 11−12.VI.2004, L.T.P. Nguyen; **Hoa Binh:** Pa Co, Mai Chau, 20°44'N, 104°55'E [1 female, alt. ca 1000 m, 27.VI.2001; 2 females, alt. ca 1000 m, 22−23.IV.2002, T.V. Hoang; 1 female, alt. ca 1350 m, 28.VIII.2006, L.T.P. Nguyen, F. Saito & J. Kojima]; **Vinh Phuc:** 1 female, Tam Dao NP, 21°32'N, 105°37'E, alt. ca 800 m, 2.VII.2003, L.T.P. Nguyen.

#### Remarks on distribution.

This species could be placed in the “*Stenopolistes*” group and has been recorded from Delhi in India and North Vietnam [Son La (Nguyen and Pham 2011), Ha Giang, Bac Kan, Phu Tho, Hoa Binh, Vinh Phuc (present study)]. The other two species of the “*Stenopolistes*” group occurring in Vietnam, *Polistes nigritarsis* Cameron and *Polistes khasianus* Cameron, similarly have such the disjunct distribution records, which are probably due to lack of intensive field works in the areas in the southern slope and western part of the eastern slope of the Himalaya.

### Polistes (Polistella) japonicus

de Saussure, 1858

http://species-id.net/wiki/Polistes_japonicus

Polistes japonicus de Saussure, 1858: 260, female, “le Japon” [lectotype in the Museum d’Histoire Naturelle, Géneve].

#### Material examined.

Northeastern provinces: **Bac Kan**: 4 females, Kim Hy NP, Na Ri, 21°15'N, 106°06'E, alt. ca 270 m, 3−4.VIII.2012, J. Kojima, H. Nugroho et al.; **Bac Giang**: 2 females, Thanh Son, Son Dong, 21°13'N, 106°45'E, alt. ca 300 m, 1.VII.2010, P.H. Pham; Tay Yen Tu NP, Son Dong, 21°21'N, 106°11'E, P.H. Pham [2 females, alt. ca 200−300 m, 3.VII.2010; 1 female, alt. 150 m, 2.VII.2010]; 1 female, Khe Ro, Son Dong, 17.V.2013, D.D. Tran. Other provinces: **Son La:** 1 female, 20°50'37"N, 104°40'17"E, alt. ca 950m, Nong Truong, Moc Chau, 2.VII.2013, D.D. Nguyen; **Lang Son:** 1 female, Bac Son, 21°54'N, 106°19'E, 1.VII.2003, L.X. Truong. Other provinces: **Phu Tho:** 1 male, 6 females, Xuan Son NP, 21°10'N, 104°58'E, alt. ca 200−600 m, 13−16.VI.2004, L.T.P.Nguyen; 1 female, Xuan Dai, Tan Son, 20.V.2011, P.H. Pham; **Ninh Binh:** 1 male, 3 females, Cuc Phuong NP, 20°19'N, 105°37'E, 7−9.V.2002, T. V. Hoang; **Thanh Hoa:** 1 male, 2 females, Lung Cao, Ba Thuoc, 20°28'N, 105°10'E, alt. ca 500 m, 12.VI.2003; 1 female, Hon Can, Van Xuan, Thuong Xuan, 23−24.VIII.2012, L.T.P. Nguyen & T.V. Hoang; **Nghe An:** Mon Son, Con Cuong 18°56'N, 104°56'E [2 males, 2 females, 22−24.VII.2004, L.T.P. Nguyen; 1 female, 9.VIII.2002; 3 females, 11.VIII.2002; 2 females, 13.IX.2005]; 1 female, Pu Mat NP, 19°6'N, 104°44'E, 26.VII.2004, L.T.P. Nguyen; 1 female, Chau Cuong, Quy Hop, 19°21'N, 105°6'E, 14−19.VII.2004, H.X. Le; 1 female, Chau Thanh, Quy Hop, 19°23'N, 105°2'E, 16.VII.2004, H.X. Le; 1 female, Co Phat, Con Cuong, 18°53'N, 104°52'E, ca 200 m, 22.VII.2006, ISD−c; 1 female, Tuong Duong, Con Cuong, 19°20'N, 104°34'E, 12.VII.2006; **Ha Tinh:** 4 males, 1female, Son Tay, Huong Son, 18°27'N, 105°20'E, 19−27.V.2004, L.T.P. Nguyen; 1 female, Rao An, Huong Son, 18°34'N, 105°10'E, 20.IV.1998, L.D. Khuat.

#### Remarks on distribution.

In Vietnam, this species has been recorded in the provinces of Ha Giang, Lai Chau, Hoa Binh, Ha Noi, Thua Thien Hue (Nguyen and Khuat 2003), Phu Tho, Hai Phong (Nguyen et al. 2005), Quang Binh, Quang Tri, Thua Thien Hue, Quang Nam (Nguyen and Ta 2008), Son La (Nguyen and Pham 2011), Bac Kan, Lang Son, Bac Giang, Ninh Binh, Thanh Hoa, Nghe An, Ha Tinh (present study), showing that the species is widely distributed in Vietnam except for southern provinces. This species could occur widely in eastern parts of subtropical and temperate Asia, from Vietnam, through eastern parts of continental China, to Korea and Honshu Island of Japan; its closely related species, *Polistes formosanus* Sonan may co-occur with this species in Taiwan and only *Polistes formosanus* is known to occur in the Nansei Islands (Saito et al. 2007).

### Polistes (Polistella) sagittarius

de Saussure, 1853

http://species-id.net/wiki/Polistes_sagittarius

Polistes sagittarius de Saussure, 1853: 56, female, “Les Indes−Orientales, la Chine” [syntypes in the Museum d’Histoire Naturelle, Genève, and The Natural History Museum, London].

#### Material examined.

Northeastern provinces **Ha Giang:** 1 male, Tung Ba, Vi Xuyen, 24.VI.2013, T.V. Nguyen; **Bac Kan:** 2 females, Kim Hy NP,Na Ri, alt. ca 600−700 m, 22°19'N, 105°54'E, 4.VIII.2012, L.T.P. Nguyen et al.; **Bac Giang:** 1 female, Thanh Son, Son Dong, 21°13'N, 106°45'E, 7.VII.2010, D.D. Tran. Other provinces: **Hai Phong:** 1 female, Cat Ba NP, Cat Hai, 20°43'N, 107°04'E, alt. ca. 30 m, 26.VII.2013, L.T.P. Nguyen & D.D. Nguyen;Other provinces: **Lao Cai:** 1 female,Ta Chai, Bac Ha, 22°31'N, 104°17'E, 26.VI.2008, L.T.P. Nguyen & P.H. Pham; **Nghe An:** 1 female, Mon Son, Con Cuong, 18°56'N, 104°56'E, 27.VII.2004, L.T.P. Nguyen; 1 female, Khe Bo, Con Cuong, 19°03'N, 104°43'E, alt. ca 120 m, 25−28.IV.1998, J.M. Carpenter; **Gia Lai:** 3 females, Ia Pal, Chu Se, 13°39'N, 108°08'E, alt. ca 370 m, 20−21.VII.2012, L.T.P. Nguyen; **Dak Lak:** 1 female, Buon Ho, 121°59'N, 108°14'E, alt. ca 770 m, 23.VII.2012, L.T.P. Nguyen; **Binh Duong:** 2 females, Binh Hoa, Thuan An, 10°54'N, 106°43'E, 20.VII.2002, L.D. Khuat.

#### Remarks on distribution.

This species is widely distributed in southern Asia with subtropical and tropical climates, from northwestern India in the west, through continental southeast Asia, to Palawan in the Phillippines, Sulawesi and Flores in the Lesser Sunda Islands in the east. Recorded widely in the provinces of Ha Giang, Lai Chau, Vinh Phuc, Ha Tay (Nguyen and Khuat 2003), Son La, Hoa Binh (Nguyen and Pham 2011), Lao Cai, Bac Kan, Bac Giang, Hai Phong, Nghe An, Gia Lai, Dak Lak, Binh Duong (present study), this species may occur throughout Vietnam.

### Polistes (Polistella) strigosus

Bequaert, 1940

http://species-id.net/wiki/Polistes_strigosus

Polistes strigosus Bequaert, 1940: 269, female, male “Wong-Sa-Shui, South Kwangsi, China” [holotype female in the Museum of Comparative Zoology, Cambridge, USA].

#### Material examined.

Northeastern provinces: **Ha Giang:** 1male, 6 females, Cao Bo, Vi Xuyen, 22°44'N, 104°54'E, 21.X.2006, L.D. Khuat; **Cao Bang:** 1 female, Phi Oac NR, Thanh Cong, Nguyen Binh, 22°35'34"N, 105°51'25"E, alt. ca 1035 m, 7−10.V.2013, T.V. Hoang; **Lang Son:** 1 female, Nong truong Thai Binh, Dinh Lap, 16.V.2013, D.D. Tran; **Bac Giang**: Son Dong, P.H. Pham [1 male, 2 females, Thanh Lam, 21°20'N, 106°19'E, alt. ca 120 m, 4.VII.2010; 1 female, Tay Yen Tu NP, 21°24'N, 106°56'E, alt. ca 150m, 2.VII.2010]. Other provinces: **Vinh Phuc:** 1 female, Tam Dao NP, 21°27'N, 105°39'E, alt. ca 1200 m, 2.VII.2003, L.T.P. Nguyen; **Ha Noi:** 1 female, Khat Thuong, Ba Vi, 21°5'N, 105°22'E, alt. ca >100 m, 16.VIII.2006, ISD−c; 1 female, Yen Bai, Van Hoa, Ba Vi, 21°1'N, 105°27'E, 15.VIII.2006, ISD−c; **Nghe An:** 3 females, Chau Cuong, Quy Hop, 19°21'N, 105°6'E, 14−19.VII.2004, H.X. Le; 1 female, Pha Lay, Mon Son, Con Cuong, 18°56'N, 104°56'E, 9.VIII.2002, ISD−c; **Ha Tinh:** 1 male, 1 female, Son Tay, Huong Son, 18°27'N, 105°21'E, 19−27.V.2004, L.T.P. Nguyen; Ta Rut, Dakrong, Quang Tri 16°25'N, 106°59'E [4 females, 17.VII.2004; 9 females, alt. ca 400−500 m, 17.VII.2004], ISD−c.

#### Remarks on distribution.

The following three subspecies are currently recognized in *Polistes strigosus*: the nominotypoical subspecies known to occur in Laos, China and Taiwan; *minimus* Bequaert, 1940 distributed in Nepal, Malaysia (Sabah) and the Philippines; and *atratus* Das and Gupta, 1984 in India. The color form from Vietnam agrees with non of the above-mentioned subspecies. It has the head reddish brown, mesosoma dark yellowish brown with metanotum and propodeum dark yellow, metasomal terga I−III dark yellow, and the other metasomal terga brownish black (in some specimens, all metasomal terga dark yellow). This species is widely recorded from the provinces of Hai Phong (Nguyen et al. 2005), Quang Binh, Quang Tri, Thua Thien Hue (Nguyen and Ta 2008), Hoa Binh (Nguyen and Pham 2011), Ha Giang, Cao Bang, Lang Son, Vinh Phuc, Bac Giang, Ha Noi, Nghe An, Ha Tinh (present study), and may occurs in eastern parts of Vietnam north of the Hai Van Pass.

### Polistes (Polistella) brunetus

Nguyen & Kojima sp. n.

http://zoobank.org/CFB91293-9882-43F9-97EB-85472BB3FCB4

http://species-id.net/wiki/Polistes_brunetus

#### Type−locality.

Vietnam, Bac Kan: Kim Hi National Park, Lang San, Na Ri, 22°19'N, 105°54'E, 600−700 m.

#### Type specimens.

Holotype, female, pinned (deposited in the Institute of Ecology and Biological Resources, Hanoi). Original label: “VIETNAM, Kim Hy NP, Lang San, Na Ri, Bac Kan, 22°19'N, 105°54'E, 600−700 m, Nest#VN−NE2012−P−02, 4.viii.2012, J.Kojima, L.T.P. Nguyen & IED−c”. Paratypes: **Bac Kan:** 2 females & 1 male, 1 female (Natural History Collection at Ibaraki University, Mito), same data as holotype.

#### Other material examined.

Northeastern provinces: **Ha Giang:** 1 female, Tung Ba, Vi Xuyen, 3.VII.2013, T.V. Nguyen; **Lang Son:** 1female, Bac Son, 21°54'N, 106°19'E, 1.VII.2003, L.X. Truong; **Bac Giang**: Son Dong, P.H. Pham [2 females, Tay Yen Tu NP, 21°24'N, 106°54'E, alt. ca 200−300 m, 3.VII.2010; 1 male, 1 female, Thanh Lam, 21°19'N, 106°20'E, alt. ca 120 m, 4.VII.2010]; 1 female, Khe Ro, Son Dong, 17.V.2013, D.D. Nguyen. Other provinces: **Hai Phong:** 1 male, Cat Ba NP, 20°42'N, 107°4'E, 15−18.VIII.2003, L.T.P. Nguyen; **Phu Tho:** Xuan Son NP, 21°8'N, 104°59'E, alt. ca 400−600 m [2 females, 11.VI.2004; 1 female, 13.VI.2004; 1 female, 16.VI.2004], L.T.P. Nguyen; **Hoa Binh:** 1 male, Lac Thinh, Yen Thuy, 20°24'N, 105°33'E, 30.IV.2002, L.D. Khuat; 1 female, Da Phuc, Yen Thuy, 20°24'N, 105°33'E, 27.VII.2000, L.X. Truong; **Ninh Binh:** 1 male, 2 females, Cuc Phuong NP, 20°18'N, 105°37'E, 7.V.2002, T. V. Hoang; **Thanh Hoa:** 1 female, Bat Mot, Thuong Xuan, Xuan Lien NP, 19°59'N, 104°59'E, ca 705 m, 23.IV.2013, L.T.P. Nguyen; **Nghe An**: 1 female, Chau Cuong, Quy Hop, 19°21'N, 105°6'E, 14−19.VII.2004, H.X. Le; **Thua Thien Hue:** 1 female, Phu Loc, Bach Ma NP, 30.V.2001, L.D. Khuat.

#### Diagnosis.

This species can be distinguished from the other *Polistes (Polistella)* species by the following combination of characters: pronotum with dense and coarse punctures, their edges forming reticulation; metasomal sternum II in lateral view swollen ventrally in anterior half; sternum IV with two long parallel longitudinal ridges medially (this character also occurs in *Polistes japonicus*); proximal margin of the penis valve of the male genitalia in lateral view produced ventrally at proximoventral to form a small tooth.

#### Description.

**Female.** Body length 15–16.5 mm (holotype: about 16 mm); fore wing length 16–17 mm (holotype: about 16.5 mm).

**Figures 2–9.**
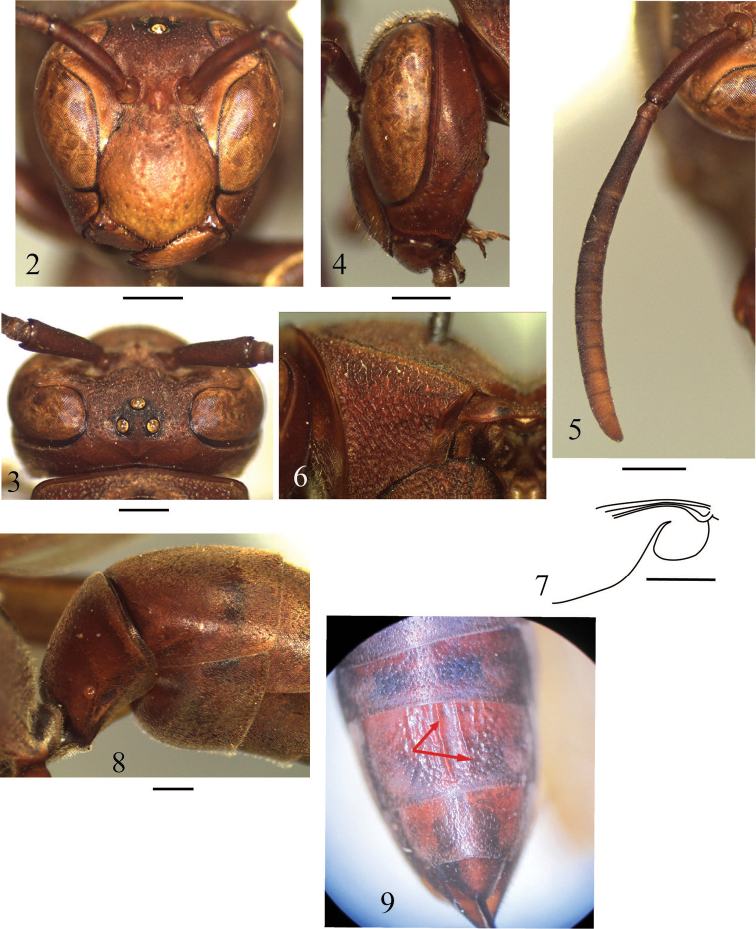
*Polistes (Polistella) brunetus* sp. n., female. **2–5** Head **2** Frontal view **3** Vertex in dorsal view **4** Lateral view **5** Right antenna **6** Pronotum in lateral view **7** Jugal lobe of left hind wing **8** Metasomal segments I and II, lateral view **9** Metasomal segment II–VI. Scale 1 mm.

Head in frontal view about 1.1 times as wide as high (Fig. 2); in dorsal view weakly swollen laterally behind eyes, then narrowed posteriorly, with posterior margin shallowly and broadly emarginate. Vertex slightly raised in area among ocelli, slightly sloped down behind posterior ocelli towards occipital carina; POD:OOD = about 1:1.7; POD about 1.2 times Od (Fig. 3). Gena, in lateral view about 0.8 times as wide as eye (Fig. 4); occipital carina fine, evanescent in ventral one third of gena. Inner eye margins weakly convergent ventrally, in frontal view about 1.1 times further apart from each other at clypeus than at vertex (Fig. 2). Antennal sockets closer to inner eye margin than to each other; anterior tentorial pit slightly further apart from antennal socket than from inner eye margin; interantennal space weakly raised. Clypeus in frontal view as wide as high, produced ventrally into blunt angle; in lateral view weakly swollen anteriorly (Fig. 3); length of lateral margin of clypeus lying along inner eye margin longer than diameter of antennal socket and about as long as the length of malar space. Antenna (Fig. 5): scape more than 3 times as long as its maximum width; flagellomere I about 3 times as long as its maximum width, about 1.2 times as long as the length of flagellomeres II and III combined; flagellomere II and III longer than wide; terminal flagellomere bullet−shaped, about 1.4 times as long as its basal width.

Pronotal carina sharply raised, produced dorsally into thin lamella in dorsal part, slightly sinuate backward on lateral side, reaching ventral corner of pronotum. Mesocutum weakly convex, about 0.9 times as long as wide between tegulae; anterior margin broadly rounded. Scutellum convex, slightly concave medially. Metanotum weakly convex, disc nearly flat but strongly depressed anterior margin. Propodeum short; posterior face widely (about half the maximum width of propodeum) and shallowly excavated medially, more or less smoothly passing into lateral faces; propodeal orifice elongate, about 1.8 times as long as wide (measured at widest part), somewhat narrowed in dorsal half. Wings hyaline, jugal lobe of hind wing rounded (Fig. 7).

Metasomal tergum I short and thick, about 0.8 times as long as its apical width, in lateral view abruptly swollen dorsally just behind basal slit for reception of propodeal suspensory ligament; corner between anterior and dorsal faces bluntly angled (Fig. 8). Sternum II in lateral view swollen ventrally in smoothly curved line in anterior half, then ventral margin nearly straight line parallel to ventral margin of the tergum.

Clypeus with scattered large punctures, each bearing sharply pointed golden bristle; tomentum on clypeus medially restricted in dorsal one-fourth of clypeus, laterally extending ventrally. Mandible with several small and shallow punctures at base and deep punctures at anterior margin. Frons covered with deep punctures. Vertex and gena with sparse small and shallow punctures; area around ocelli smooth; ventral one third of gena with coarse punctures. Pronotum with dense, coarse punctures, their edges forming reticulation (Fig. 6). Mesocutum densely with coarse flat-bottomed punctures; punctures on scutellum and metanotum dense coarser but smaller than those on mesoscutum. Mesepisternum with dense coarse well−defined punctures in posterodorsal part (punctures in dorsal margin similar to those on pronotum), scattered punctured in anteroventral part; border between posterodorsal and anteroventral parts indistinct. Dorsal metapleuron with striae and shallow large punctures; ventral metapleuron with sparse strong punctures. Propodeum with strong transverse striae; lateral face with sparse ill−defined punctures. Metasomal segements covered with minute punctures in addition to scattered small punctures (stronger and larger on sterna) except sternum IV with two long medial parallel longitudinal ridges along sternum and several shorter ridges on each side of the long one ended by large shallow punctures (Fig. 9), area between paired longitudinal ridges smooth; sternum II−IV each with a stuff of long hair at apical margin, sternum V and VI entirely covered with long hairs.

Dark brown; following parts yellow to orange-yellow: clypeus except apical black margin, mandible except a black spot at base and apical margin, and narrow band along inner eye margin extending from bottom of frons to middle of eye emargination; following parts black: area around ocelli, apical margin and a longitudinal line along lateral faces and at the middle of propodeum, spot on valvula, mid and hind coxae and trochanters beneath.

**Male.** Body length about 13.5–15.5 mm; fore wing length about 15.5–16.5 mm.

**Figures 10–17.**
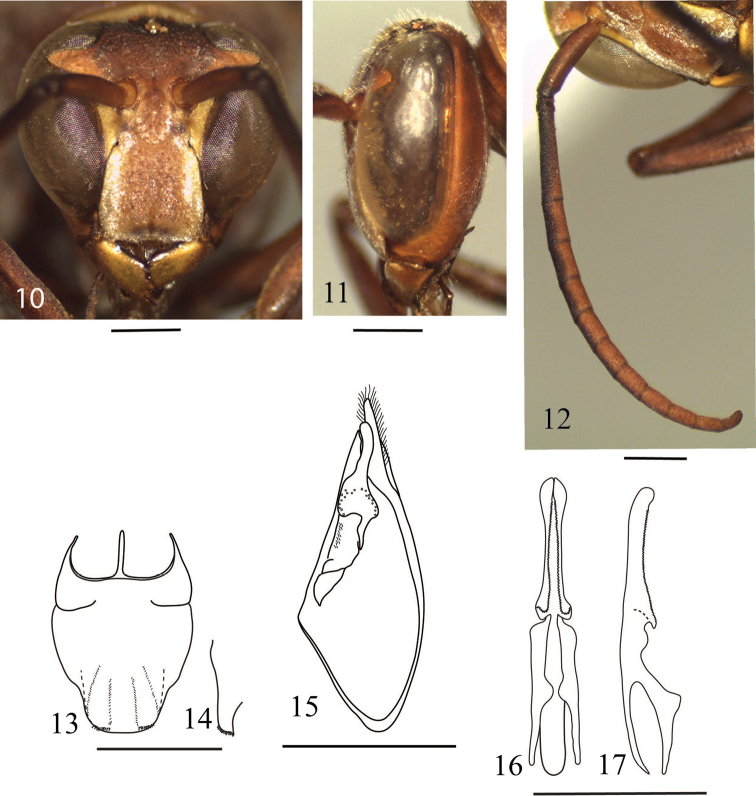
*Polistes (Polistella) brunetus* sp. n., male. **10–11** Head **10** Frontal view **11** Lateral view **12** Right antenna **13–14** Terminal sterna **13** Ventral view **14** Apical part, lateral view **15** Inner aspect of paremere with digitus and volsella **16–17** Aedeagus **16** Ventral view **17** Lateral view. Scale 1 mm.

Like female, but differing from the latter as follows: head about 1.2 times as wide as high in frontal view (Fig. 10); eye strongly swollen laterally; inner eye margins about as long from each other at vertex as at clypeus; gena in lateral view about half as wide as eye (Fig. 11), with weakly raised blunt ridge running along posterior margin of eye; clypeus in frontal view about as wide as high (Fig. 10), only slightly produced ventrally, evenly and slightly convex apically, in lateral view weakly convex in dorsal part (Fig. 11). Antenna (Fig. 12) slenderer than in female; scape short, about 2.8 times as long as its maximum width; flagellomere I longer than length of flagellomeres II and III combined; flagellomeres II and III each longer than wide; terminal flagellomere elongate, slightly curved, about 2.5 times as long as its basal width. Metasomal sternum VII depressed medially (Fig. 13), without tubercle (Fig. 14).

Body surface sculpture as in female, but clypeus without large punctures and densely covered with long golden hairs and with a faint longitudinal ridge medially.

Genitalia in general as that of *Polistes* species, with the following specific characters: digitus in inner aspect of paramere (Fig. 15) about 3.2 times as long as wide (measured at widest part), distinctly swollen near base, gradually narrowed apically to mid−length, then slightly swollen towards the rounded apex; aedeagal penis valves (Figs 16−17) slightly longer than aedeagal basal apodeme, in ventral view narrowest near mid−length, weakly swollen proximally from mid−length then strongly swollen and distinctly produced laterally near proximal margins, in lateral view slightly thickened in proximal one fourth and with dorsal margin weakly and smoothly sinuate, with proximoventral corner produced into abuse angle (Fig. 17); ventral margins of penis valves finely serrated along entire length.

Color and marking pattern similar to female, but more extensively marked with yellow as follows: clypeus except a broad longitudinal median band, narrow long band on gena along posterodorsal margin of eye, antennal flagellomeres beneath, narrow band along pronotal lamella, narrow band on basal metanotum, paired longitudinal lines on lateral face of propodeum, valvulae, narrow band at apical margin of tergum I−III and sternum II and III; more extensively black marked as follow: two longitudinal bands at lateral margin on mesoscutum, a wide band on basal margin of tergum I and II (sometimes on tergum III); spot at upper corner of meseposternum (closed to dorsal part of metapleuron); propodeum and legs more black.

#### Etymology.

The specific name, *brunetus*, is a Latin adjective, referring to the brown body color.

#### Distribution.

Known only from localities in northern Vietnam listed above.

## Nests

### *Polistes (Polistella) mandarinus* de Saussure, 1853

**Figures 18–20.**
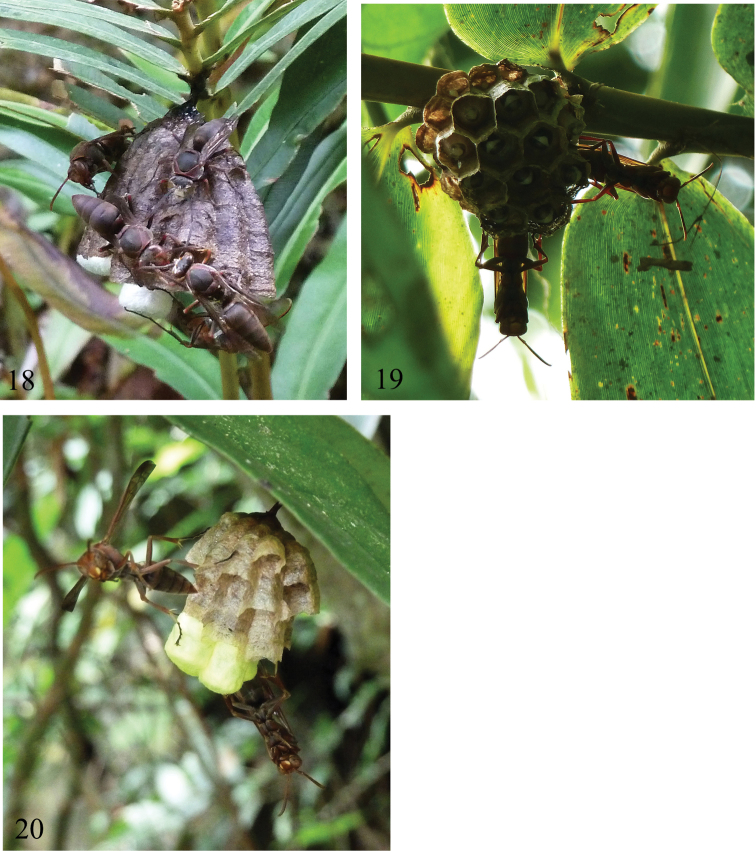
Nests. **18** *Polistes mandarinus* **19** *Polistes brunetus* sp. n. **20** *Polistes delhiensis*.

Hou et al. (2012) described the nest of this species based on the nests observed in Tibet, with light ferruginous brown (juggling from the figures). A nest (#VN−NE2012−P−02) (Fig. 18) that we collected, together with 3 females and 11 males, at Phi Oac NR, Cao Bang Province has similar features of that described by Hou et al. (2012) although it differs in coloration. Our nest has 19 cells and had produced more than ten adult wasps. Its structural and color characters are as follows: **Comb** “paper”−like in texture, made mainly of long fine plant fibers and wasp adult oral secretion, more or less uniformly dark greysish−brown in cell walls, suboval (about 30 mm × 20 mm) in view from side of cell opening, expanded excentrically from the single terminal petiole, with surface corresponding to cell bottom weakly convex; **Petiole** single, terminal, attached to the border between bottoms of the first two cells, 2.5 mm long and 1.2 mm × 1.5 mm thick at the mid−length, with thin central core of plant fibers, enlarged strictly with adult oral secretion, blakish brown and lustrous, secretion coat widely expanded on comb back around the petiole and on substrate in thin film holding the fern vain; **Cells** generally arranged in regular rows, pentagonal at open end when surrounded by other cells, with free margins rounded, each cell weakly expanded towards open end, 5.3 mm × 5.6 mm (range 5.0 mm × 5.4 mm – 5.8 mm × 5.9 mm; n=17) wide at open end, 3.4 mm (range 3.1−3.8 mm; n=11) wide at bottom and 19 mm (range 15−22.5 mm; n=13) deep in cells containing pupae or having produced adult, cell wall about 1.12 mm thick; **Cocoon cap** white, produced beyond rim of cell by 0.5−4.5 mm, slightly domed.

### *Polistes (Polistella) brunetus* Nguyen & Kojima, sp. n.

A pre−emergence stage (before any adult wasps’ emergence) nest (#VN−NE2012−P−02) (Fig. 19) was collected, together with 4 adult females, at Kim Hy NP, Bac Kan Province. The nest was attached to a rattan shoot, at about 2.5 m above the ground, and has 26 cells, with the fifth (=last) instar larvae as the oldest immature (for immature composition, see Fig. 19). The fifth instar larvae were artificially fed with fresh hornet (*Vespa*) eggs, and one of them successfully spun the cocoon. The structural and color characters are as follows: **Comb** “paper”−like in texture, made mainly of long fine plant fibers, usually with 2−3 mm wide horizontal stripes of different colors (pale gray to gray and pale brownish−gray) in cell walls, subcircular (about 30 mm × 25 mm) in view from side of cell opening, expanded concentrically from the single petiole, with surface corresponding to cell bottom weakly convex; **Petiole** single, central, attached to the border between bottoms of the first two cells, 3.8 mm long and 1.2 mm × 1.2 mm thick at the mid−length, with thin central core of plant fibers, enlarged strictly with oral secretion of adults, brown and lustrous, secretion coat widely expanded on comb back around the petiole and on substrate in about 8 mm × 9 mm subcircular thin film; **Cells** generally arranged in regular rows, pentagonal at open end when surrounded by other cells, with free margins rounded, each cell weakly expanded towards open end, 6.3 mm × 6.7 mm (range 6.0 mm × 6.1 mm – 7.1 mm × 7.2 mm; n=5) wide at open end, 5.1 mm (range 4.9−5.4 mm; n=5) wide at bottom and 18.5 mm (range 17−19.5 mm; n=5) deep in cells containing mature larvae, cell wall about 0.09 mm thick; **Cocoon cap** white, slightly domed.

### *Polistes (Polistella) delhiensis* Das & Gupta, 1989

A pre−emargence stage (before any adult wasps’ emergence) nest (# VN−NE2012−P−04) (Fig. 20) examined was collected, together with 3 females, at Kim Hy NP, Bac Kan province. The nest was attached to a broad leaf, at about 1 m above the ground, and has 19 cells, which contained the pupae as the oldest immatures. The structural and morphological characters are as follows: **Comb** “paper”−like in texture, made mainly of long fine plant fibers mixed with adult oral secretion, pale brown to brown in cell walls, subcircular (about 19 mm × 17 mm) in view from side of cell opening, expanded concentrically from the single petiole, with surface corresponding to cell bottom slightly convex; **Petiole** single, central, attached to the border between bottoms of the first two cells, 2.5 mm long and 0.5 mm × 0.7 mm thick at the mid−length, with thin central core of plant fibers, enlarged strictly with adult oral secretion, dark brown and lustrous, secretion coat widely expanded on comb back around the petiole and on substrate in about 2.5 mm × 5 mm thin film; **Cells** generally arranged in regular rows, pentagonal at open end when surrounded by other cells, with free margins rounded, each cell weakly expanded towards open end, 4.4 mm × 4.5 mm (range 4.3 mm × 4.4 mm – 4.5 mm × 4.5 mm; n=4) wide at open end, 3.2 mm (range 3.0−3.3 mm; n=4) wide at bottom and 14 mm (range 13.5−14.5 mm; n=4) deep in cocooned, cell wall about 0.06 mm thick; **Cocoon cap** pale greenish−yellow, prominently produced beyond rim of cell by 5−6 mm, slightly domed.

## Key to species of *Polistes (Polistella)* of northeastern Vietnam

The characters given in the key are applicable to both sexes unless when specified.

**Table:** 

1	Metasomal sternum II basally strongly swollen, in lateral view bulging anteriorly (Fig. 21)	2
–	Metasomal sternum II gradually swollen posteriorly, in lateral view with ventral margin weakly and smoothly curved (Fig. 8)	3
2	Clypeus in lateral view only weakly convex anteriorly (Fig. 22). Disc of scutellum nearly flat, in lateral view smoothly passing from dorsal margin of mesoscutum (Fig. 24). Male clypeus as wide as high	*Polistes dawnae* Dover & Rao
–	Clypeus in lateral view distincly convex (Fig. 23). Disc of scutellum convex (Fig. 25). Male clypeus about 1.1 times as wide as high	*Polistes mandarinus* de Saussure
3	Medium-sized wasps; fore wing length 10.5−14.5 mm. Jugal lobe of fore wing much reduced. Marginal cell of fore wing with dark spot at apex	*Polistes delhiensis* Das & Goupta
−	Large-sized wasps; fore wing length 15.5−18 mm. Jugal lobe of fore wing large, rounded. Marginal cell of fore wing without dark spot	4
4	Pronotum with strong striation (Figs 26, 27) Female metasomal sterna without longitudinal ridges	5
–	Pronotum with weak striation weak or absent. Female metasomal sternum IV medially with paired longitudinal ridges (Fig. 9)	6
5	Pronotal striation somewhat irregular (Fig. 26), spaces between striae densely and distincly punctured. Dorsal surface of pronotum smoothly curved down to the lateral surface. Male metasomal sternum VII without tubercle. Clypeus, mesoscutum, metasomal segments III−VI entirely black	*Polistes sagittarius* de Saussure
–	Pronotal striation regular and very strong (Fig. 27). Border between dorsal and lateral surfaces of pronotum distinctly angled. Male metasomal sternum VII with weak tubercle (Fig. 28). Clypeus, mesoscutum, metasomal segments II−VI etirtely brown	*Polistes strigosus* Bequaert
6	Pronotum with dense, coarse punctures, their edges forming reticulation (Fig. 6). Disc of scutellum convex. Metasomal sternum II in lateral view convex ventrally in anterior half. Anterior margin of male clypeus nearly straight (Fig. 10). Metasomal terga brown, with some black marks. Wings hyaline	*Polistes brunetus* Nguyen & Kojima, sp. n.
–	Pronotum with sparse, small punctures. Disc of scutellum hardly convex. Metasomal sternum II in lateral view weakly convex ventrally in the anterior two thirds. Anterior margin of male clypeus rounded. Metasomal terga yellow with dark brown and/or black bands. Wings infuscate	*Polistes japonicus* de Saussure

**Figures 21–28.**
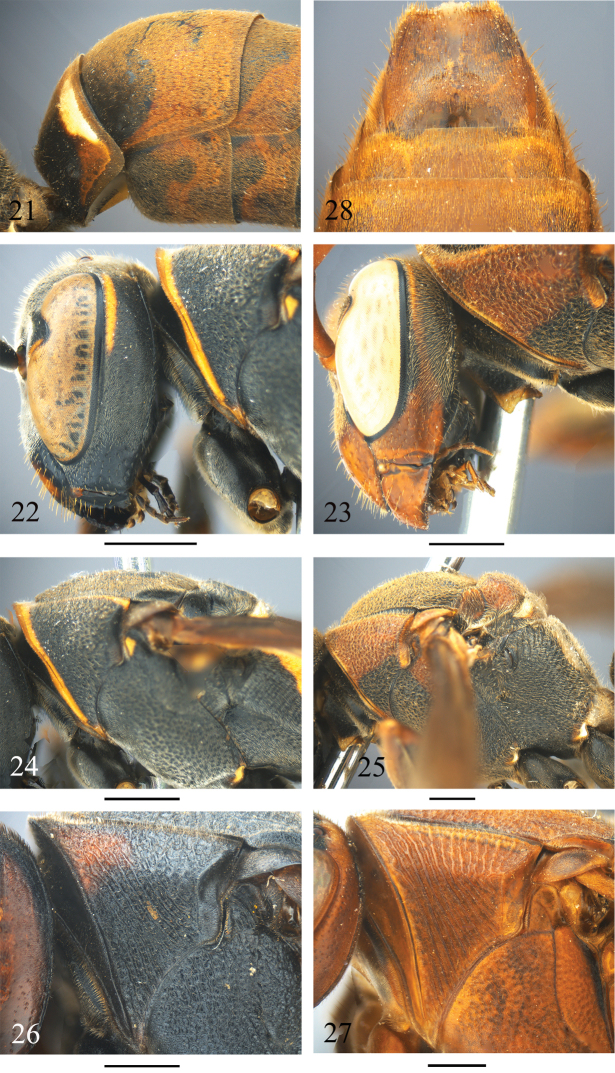
*Polistes* species characters. **21, 23, 25** *Polistes mandarinus*: **21** Metasomal segment I and II, lateral view **23** Head, lateral view **25** Mesosoma, lateral view **22, 24** *Polistes dawnae*: **22** Head, lateral view **24** Mesosoma, lateral view **26** P. sagittarius, pronotum, lateral view **27–28** *Polistes strigosus*: **27** Pronotum, lateral view **28** Metasomal sternum VII, ventral view. Scale 1 mm.

## Conclusion

Compared with the *Polistella* fauna in mountainous areas of northern (mainly northwestern) Vietnam, where 14 *Polistella* species have been recognized (Nguyen et al. 2011), *Polistella* fauna in northeastern Vietnam, with only seven species, is poorer even though the environmental, especially climatic conditions, in northeastern Vietnam are expected to be more diverse and hence to harbor richer fauna in terms of number of species than in mountainous areas of northern Vietnam. On the other hand, however, in contrast to the fact that all the 14 *Polistella* species occurring in mountainous areas in northern Vietnam may belong to a possible monophyletic species group that is characterized by a basally strongly swollen second metasomal sternum and shows ditribution pattern of so−called “Himalayan Corridor origin”, seven species recognized in northeastern Vietnam are comprised of at least three species groups, thus they are more diverse phylogenetically than those of mountainous areas of norther Vietnam. Namely, other than *Polistes dawnae* and *Polistes mandarinus* in the species group characterized by a basally strongly swollen second metasomal sternum, *Polistes delhiensis* belongs to so-called “*Stenopolistes*” group, the species belonging to which are distributed in tropical and subtropical continental Asia, so-called Sunda Land (Malay Peninsular, Sumatra and Borneo) and also in Papuan Region, including Pacific Islands. The last group, including the four species recognized in northeastern Vietnam, *Polistes japonicus*, *Polistes sagittarius*, *Polistes strigosus* and *Polistes brunetus*, is the *Polistes sagittarius* group of Carpenter (1996), which is rather ill-defined and known to be widely distributed in Oriental Region and East Asia.

## Supplementary Material

XML Treatment for Polistes (Polistella) dawnae

XML Treatment for Polistes (Polistella) mandarinus

XML Treatment for Polistes (Polistella) delhiensis

XML Treatment for Polistes (Polistella) japonicus

XML Treatment for Polistes (Polistella) sagittarius

XML Treatment for Polistes (Polistella) strigosus

XML Treatment for Polistes (Polistella) brunetus
